# Vascular Calcification in Chronic Kidney Disease: The Role of Inflammation

**DOI:** 10.1155/2018/4310379

**Published:** 2018-08-13

**Authors:** Kerstin Benz, Karl-Friedrich Hilgers, Christoph Daniel, Kerstin Amann

**Affiliations:** ^1^Department of Nephropathology, Friedrich-Alexander University (FAU) Erlangen-Nürnberg, Germany; ^2^Department of Nephrology and Hypertension, Friedrich-Alexander University (FAU) Erlangen-Nürnberg, Germany

## Abstract

Cardiovascular complications are extremely frequent in patients with chronic kidney disease (CKD) and death from cardiac causes is the most common cause of death in this particular population. Cardiovascular disease is approximately 3 times more frequent in patients with CKD than in other known cardiovascular risk groups and cardiovascular mortality is approximately 10-fold more frequent in patients on dialysis compared to the age- and sex-matched segments of the nonrenal population. Among other structural and functional factors advanced calcification of atherosclerotic plaques as well as of the arterial and venous media has been described as potentially relevant for this high cardiovascular morbidity and mortality. One potential explanation for this exceedingly high vascular calcification in animal models as well as in patients with CKD increased systemic and most importantly local (micro)inflammation that has been shown to favor the development of calcifying particles by multiple ways. Of note, local vascular upregulation of proinflammatory and proosteogenic molecules is already present at early stages of CKD and may thus be operative for vascular calcification. In addition, increased expression of costimulatory molecules and mast cells has also been documented in patients with CKD pointing to a more inflammatory and potentially less stable phenotype of coronary atherosclerotic plaques in CKD.

## 1. Introduction

Patients with chronic kidney disease (CKD) and chronic renal failure (CRF) develop early on in the course of the disease structural and functional alterations of the heart and the vascular tree that represent a major clinical problem in these patients. In addition, cardiovascular diseases are a major contributor to the high incidence of cardiovascular complications and particularly death from cardiovascular causes in this population [1, 2]. Apart from left ventricular hypertrophy (LVH) which is present very early in the course of the renal disease even in normotensive CKD patients structural alterations of the myocardium as well as the intra- and extracardiac arteries and veins are hallmarks of this disease. With respect to the myocardium reduced myocardial capillary supply, interstitial fibrosis and thickening of the intramyocardial arteries can be seen [3–5]. These intramyocardial structural alterations increase the susceptibility of the hypertrophied heart of CKD patients towards ischemic damage and favor the development of arrhythmias, myocardial infarction, and sudden cardiac death [6]. The pathogenesis of these myocardial alterations is certainly multifactorial, but only partly understood. Among the mechanisms that have been described for LVH and the associated myocardial alterations are the so-called classical or traditional risk factors like hypertension, hyperlipidemia, and diabetes but also CKD-specific changes such as anemia, hypervolemia, increased sympathetic activity, hyperphosphatemia, oxidative stress, and altered expression of the fibroblast growth factor 23 (FGF-32) [7, 8]. As a consequence clinical strategies to prevent or ameliorate LVH and associated structural alterations in CKD comprise prevention of anemia by normalization of the hemoglobin value (Hb), prevention of hypervolemia, and strict blood pressure control particularly with ACE-inhibitors. In experimental models of chronic renal failure blockade of the renin-angiotensin system (RAS), the endothelin (ET) system, pharmacological and mechanical inhibition of the sympathetic nervous system, and blockade of the FGF23-axis [9–13] were also shown to be successful.

**Figure 1:**
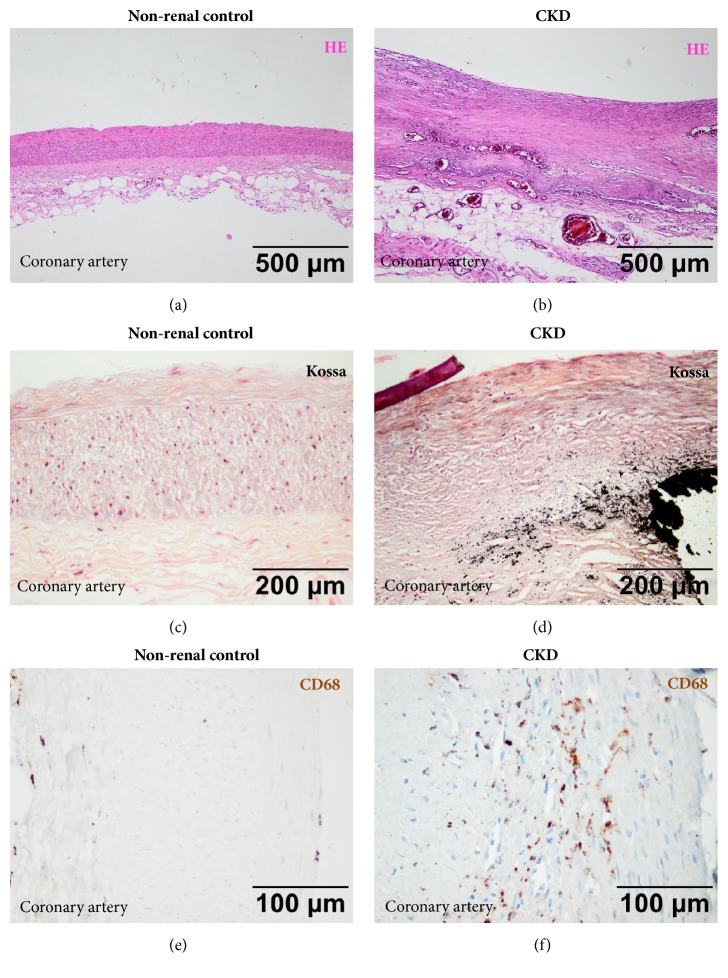
**(a-f) Representative vascular findings in nonrenal control patients (a, c, e) and patients with chronic kidney disease (CKD).** (a, b) Marked thickening of coronary arteries in CKD patients (b) compared to nonrenal controls (a). HE-stain. (c, d) Marked calcification of the arterial intima and media and atherosclerotic coronary plaques in CKD patients (d) compared to nonrenal controls (d). Von Kossa stain. (e, f) Increased number of CD68 positive macrophages in the vascular wall of CKD patients (f) compared to nonrenal controls (e). Immunohistochemistry.

In addition to the cardiac alterations specific structural changes of the extracardiac arteries and veins are present which consist of vessel thickening (Figures 1(a) and 1(b)) but more importantly of marked calcification of the arterial intima and media as well as of venous walls and atherosclerotic coronary plaques giving rise to complications such as coronary artery thrombosis and myocardial infarction [14–18]. Based on their clinical experience the group of Lindner and coworkers in 1974 [19] were the first to show that atherosclerosis in CKD patients is different from that of nonrenal patients. They particularly showed that the lesions were more advanced and the course of atherosclerosis in these patients was more aggressive. Consequently, they speculated that this may contribute to the exceedingly high cardiovascular morbidity and mortality in these patients. The functional consequences of increase vascular and plaques calcification are manifold: it leads to increased stiffness of the vessel which in the case of the arterial tree increases the cardiac afterload and impairs coronary artery perfusion [20–22]. In an autoptic study Schwarz and coworkers [23, 24] were able to confirm that indeed the coronary atherosclerotic lesions in CKD patients were more advanced than in nonrenal control patients and perhaps more importantly that particularly the calcified plaque lesions were significantly more present in CKD patients (Figures 1(c) and 1(d)). This and other findings prompted in vitro and in vivo studies investigating the underlying pathogenesis of increased plaque and vessel calcification in CKD.

## 2. Potential Pathogenesis of Advanced Vascular Calcification in CKD

Although the exact pathogenesis of increased vascular calcification in CKD is not fully understood there are some very plausible hypotheses and candidates: from the very beginning disturbances in the phosphorus (P) and calcium (Ca) metabolism [25, 26], altered expression of many factors, which are involved or regulate the mineral metabolism [27, 28], and perhaps most importantly chronic microinflammation which is present in CKD patients early on in the disease and comes along with increased CRP levels [29, 30] have been discussed as important pathogenetic factors. Particularly, early on a major pathogenetic role of P and Ca has emerged from clinical [31, 32] and experimental studies [33, 34] where a strong correlation between serum values of both P and Ca and perhaps more importantly the Ca-P product (CaxP) and cardiovascular events could be shown [35]. On the other hand, experimental manipulation with high and low phosphate diet in an animal model of chronic renal failure, i.e., the subtotally nephrectomized rat (SNX), could clearly document the deleterious cardiovascular effect of high phosphate [34]. It has also been shown in vitro that increased P and Ca levels can induce osteoblast-like changes in vascular smooth muscle cells (VSMC) via stimulation of Na-dependent P-cotransporters [36, 37]. This is reflected by de novo expression of specific marker proteins of osteogenic differentiation like cbfa-1 und osteocalcin which coincide with a change in phenotype of VSMC from a contractile to a secretory calcifying cell. In patients with CKD the expression of calcification-inhibiting proteins like Fetuin-A, MGP, beta-Glucosidase, and osteoprotegerin is reduced which may also contribute to a procalcific milieu [38, 39]. Very recently, serum calciprotein particles and circulating nanostructures, such as extracellular vesicles, have been identified as important players in the mechanism of calcification in CKD mainly by promoting cell osteochondrogenic differentiation and inflammation [40]. Vice versa, a role for inhibition of mineral crystal formation by Gla-rich protein which in vitro could prevent calciprotein particles induced calcification was postulated.

## 3. Role of Systemic and Local Inflammation in Vascular Calcification

**Figure 2:**
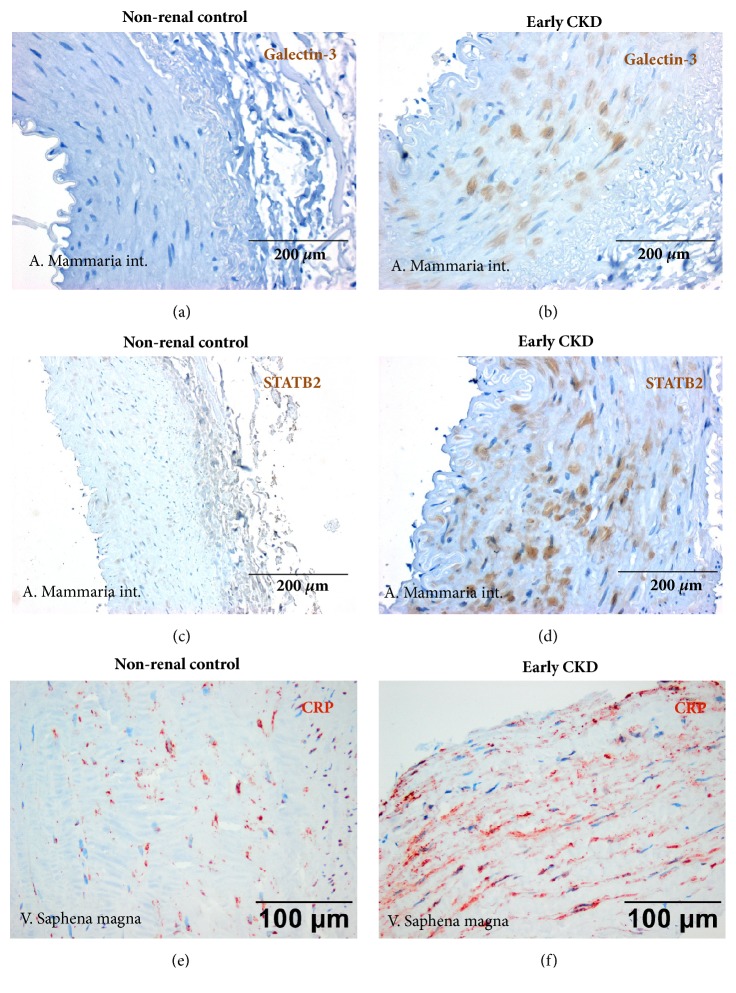
**(a-f) Representative vascular changes in nonrenal control patients (a, c, e) and patients with early chronic kidney disease (CKD).** (a, b) Increased expression of galectin-3 in arteria mammaria int. in early CKD patients (b) compared to nonrenal controls (a). Immunohistochemistry. (c, d) Marked increase in STAB2 expression in arteria mammaria int. in early CKD patients (d) compared to nonrenal controls (d). Immunohistochemistry. (e, f) Increased staining for C-reactive protein (CRP) in vena saphena magna in early CKD patients (f) compared to nonrenal controls (e). Immunohistochemistry.

The above-mentioned recent study by Viegas and coworkers [40] more emphasizes a potential role of inflammation for vascular calcification in CKD. Systemic and local effects of inflammation on vascular alterations in CKD but also in other segments of the general population are being discussed for quite some time [41, 42]. In addition, to the many excellent data on increased systemic inflammation in CKD that can be monitored by elevated inflammatory biomarkers such as CRP [30] and IL-6 [43–45] there is also experimental evidence for a major role of local inflammation in the pathogenesis of advanced atherosclerosis in renal failure [24]. In CKD patients our group could show that coronary artery plaques are not only more advanced than in nonrenal control patients but also exhibit a more inflammatory phenotype with increased local expression of proinflammatory systems such as the CD40-CD154 ligand system and also macrophages (Figures 1(e) and 1(f)) which has been associated with increased risk of atherosclerotic events [46]. In addition, we could recently show [47] that local upregulation of proinflammatory and proosteogenic molecules such as CRP, CD40, CD154, and SATB2 as well as galectin-3 is already present at early stages of CKD in various vessels (Figure 2), i.e., the A. mammaria int., the V. saphena magna, and the aorta taken at the occasion of cardiac surgery [47]. Of note, local microinflammation was even seen in the absence of major disturbances of the CaxP product and could thus be most likely regarded independent of changes in P or Ca. Local microinflammation also preceded the development of vascular calcification, a finding that may point to an early and important pathophysiological role of (pro)inflammatory changes. In addition, we recently investigated a potential role for dendritic cells (DCs) and particularly mast cells (MCs) in the pathogenesis of coronary atherosclerosis in CKD [48]. These cells might be important since a role of DCs and MCs for inflammation, immunity, and T-cell-activation in atherosclerosis [49–55] has been shown. Consequently, DCs and MCs may represent novel therapeutic targets [56] for treatment of atherosclerosis and its complications. Of note, patients with CKD are known to show clinical and laboratory features of immunosuppression with low numbers of T-cells and circulating DCs as well as impaired T-cell activation [57–59], most likely as a consequence of suppressive effects of CKD on the bone marrow and cell maturation. Using immunohistochemistry, Hueso et al. [60] investigated aortic lesions of CKD and control patients, i.e., adaptive as well as pathological intimal thickening of the aorta and fibroatheromas and found a significantly higher percentage of DCs in CKD patients compared to non-CKD controls. In contrast, in coronary arteries we found a lower number of DCs in CKD compared to nonrenal control patients. DCs were predominantly located in the shoulder, boundary, and basis subregions of plaques in good correlation with the Stary classification of atherosclerotic lesions. In addition, in the shoulder and cap region DCs positivity correlated with T-cells and macrophages potentially indicating a proinflammatory role of DCs that might be important in terms of plaque instability. It is also speculated that DCs activation by nicotine, stress, hypoxia, or CRP may stimulate the local immune response [61]. This might be of interest in view of our previous finding of an increased in situ expression of CRP in atherosclerotic plaques of CKD patients compared to controls [46]. With respect to the effect of CKD on MC number and distribution we found a tendency for more MCs in vessels from end-stage CKD patients particularly in calcified ones. This finding potentially points to a specific role of MCs compared to DCs under the condition of renal failure and particularly in vessel calcification. Detailed correlation analysis of MCs with inflammatory cells such as T-cells and macrophages inside plaques subregions revealed a positive association with T-cells in end-stage CKD specimens and a significant correlation between the MC score within the plaque and the serum C-reactive protein (CRP) levels, again suggesting a potential link of MCs to local intraplaque T-cell and macrophage activation and also to systemic inflammation.

## 4. Summary and Conclusion

Advanced stages of atherosclerotic plaques particularly with plaque and vessel calcification are specific features in patients with CKD that might contribute to the high cardiovascular morbidity and mortality in these patients. Our findings of a more inflammatory phenotype of coronary atherosclerotic plaques and particularly of a potential role of MCs for advanced atherosclerosis in CKD might be particularly important in view of recent experimental findings in the animal model of the ApoE knockout mouse [62] where pharmacological MC chymase inhibition using the protease inhibitor RO5066852 was shown to reduce atherosclerotic plaque progression and to improve plaque stability. If this novel experimental finding could be successfully translated into the human situation pharmacological MC inhibition might possibly evolve as a new therapeutic target in the treatment of atherosclerosis particularly in the advanced condition of chronic renal failure. In order to find adequate and effective treatment strategies it is tremendously important to gain further insight into the exact mechanisms of calcification under the conditions of CKD [63, 64]. It is most likely that in the near future we aim at specific treatment options for hyperphosphatemia, chronic microinflammation, and increased FGF-23 levels and may be also MC chymase inhibition in patients with CKD that might help to lower the exceedingly high cardiovascular risk in this particular population group.
